# *catena*-Poly[[bis­(3-carb­oxy-5-nitro­benzoato-κ*O* ^1^)copper(II)]-μ-1,3-di-4-pyridylpropane-κ^2^ *N*:*N*′]

**DOI:** 10.1107/S1600536809031821

**Published:** 2009-08-19

**Authors:** Laura K. Sposato, Robert L. LaDuca

**Affiliations:** a Lyman Briggs College, Department of Chemistry, Michigan State University, East Lansing, MI 48825, USA

## Abstract

In the title compound, [Cu(C_8_H_4_NO_6_)_2_(C_13_H_14_N_2_)]_*n*_, the square-planar coordinated Cu^II^ ion lies on an inversion centre and is coordinated by two protonated 5-nitro­isophthalate ligands. The Cu^II^ ions are linked into a one-dimensional coordination polymer by tethering 1,3-di-4-pyridylpropane ligands, whose central methyl­ene C atoms are situated on twofold rotation axes. The chains are oriented parallel to the *c* axis, and stack into a supra­molecular three-dimensional structure through O—H⋯O hydrogen-bonding inter­actions.

## Related literature

For some recent divalent copper dicarboxyl­ate coordination polymers containing 1,3-di-4-pyridylpropane, see: Wang *et al.* (2009 ▶).
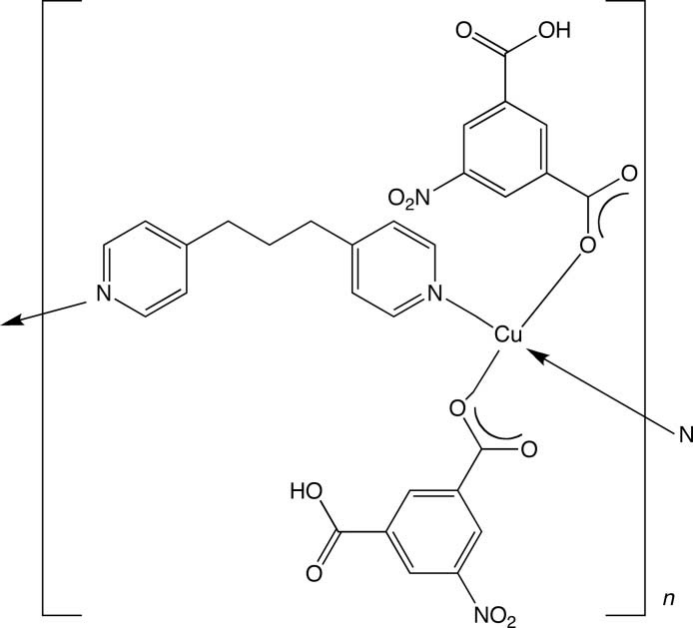


## Experimental

### 

#### Crystal data

[Cu(C_8_H_4_NO_6_)_2_(C_13_H_14_N_2_)]
*M* *_r_* = 682.05
Monoclinic, 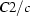

*a* = 25.2976 (8) Å
*b* = 5.3702 (2) Å
*c* = 21.3122 (7) Å
β = 115.865 (2)°
*V* = 2605.29 (15) Å^3^
*Z* = 4
Mo *K*α radiation
μ = 0.92 mm^−1^
*T* = 173 K
0.22 × 0.22 × 0.11 mm

#### Data collection

Bruker APEXII diffractometer
Absorption correction: multi-scan (*SADABS*; Sheldrick, 1996 ▶) *T*_min_ = 0.821, *T*_max_ = 0.905
10349 measured reflections
2401 independent reflections
2087 reflections with *I* > 2σ(*I*)
*R*_int_ = 0.032

#### Refinement

*R*[*F* ^2^ > 2σ(*F* ^2^)] = 0.041
*wR*(*F* ^2^) = 0.112
*S* = 1.08
2401 reflections
213 parameters
1 restraint
H atoms treated by a mixture of independent and constrained refinement
Δρ_max_ = 1.10 e Å^−3^
Δρ_min_ = −0.31 e Å^−3^

### 

Data collection: *APEX2* (Bruker, 2006 ▶); cell refinement: *SAINT* (Bruker, 2006 ▶); data reduction: *SAINT*; program(s) used to solve structure: *SHELXS97* (Sheldrick, 2008 ▶); program(s) used to refine structure: *SHELXL97* (Sheldrick, 2008 ▶); molecular graphics: *CrystalMaker* (Palmer, 2007 ▶); software used to prepare material for publication: *SHELXL97*.

## Supplementary Material

Crystal structure: contains datablocks I, global. DOI: 10.1107/S1600536809031821/tk2526sup1.cif

Structure factors: contains datablocks I. DOI: 10.1107/S1600536809031821/tk2526Isup2.hkl

Additional supplementary materials:  crystallographic information; 3D view; checkCIF report

## Figures and Tables

**Table 1:** Hydrogen-bond geometry (Å, °)

*D*—H⋯*A*	*D*—H	H⋯*A*	*D*⋯*A*	*D*—H⋯*A*
O3—H3*A*⋯O2^i^	0.85 (4)	1.86 (2)	2.668 (3)	158 (4)

Symmetry code: (i) 
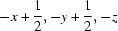
.

**Fig. 1.**
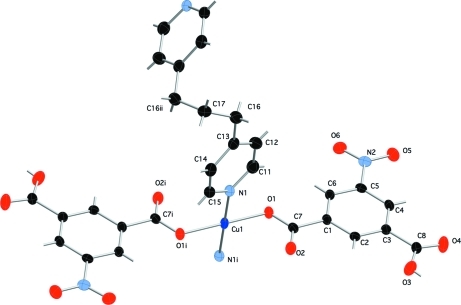
The coordination environment of (I), showing 50% probability ellipsoids and atom numbering scheme. Hydrogen atom positions are shown as grey sticks. Color codes: dark blue Cu, light blue N, orange O, black C. Symmetry codes: (i) -x, -y, -z (ii) -x, y, -z + 1/2.

**Fig. 2.**
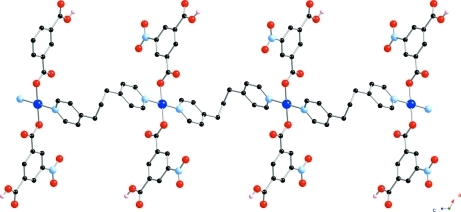
A view of the coordination polymer chain motif in (I).

**Fig. 3.**
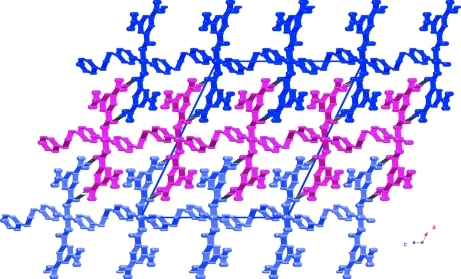
Stacking diagram for (I), viewed down the b axis. Hydrogen bonding contacts are indicated as dashed bars.

## supplementary crystallographic information

### Comment

The title compound, (I), was prepared by the hydrothermal reaction of copper nitrate, 5-nitroisophthalic acid (nip) and 1,3-di-4-pyridylpropame (dpp). Its asymmetric unit contains a copper atom on an inversion centre, one singly protonated nip (Hnip) ligand, and one-half of a dpp ligand, whose central methylene carbon atom is situated on a 2-fold axis. Operation of the inversion centre at Cu reveals a square planar coordination environment with *trans* O atom donors from protonated nip ligands and *trans* N donors from two dpp ligands (Fig. 1). The Hnip ligands are bound to Cu in a simple monodentate fashion *via* an O atom belonging to the non-protonated carboxylate terminus.

Operation of the 2-fold rotation axes indicates that dpp ligands connect neighboring Cu atoms into a one-dimensional [Cu(Hnip)_2_(dpp)]_n_ coordination polymer chain (Fig. 2), which is oriented parallel to the *c*-axis. The methylene groups within the dpp ligands adopt a *gauche-gauche* conformation, providing a Cu···Cu separation of 10.656 (4) Å. Neighboring chains aggregate by hydrogen-bonding mechanisms (Table 1) involving the hydroxyl groups of the protonated Hnip ligands and unligated O atoms of the monodentate Hnip carboxylate groups, thus forming the three-dimensional supramolecular structure (Fig. 3).

### Experimental

All starting materials were obtained commercially. A mixture of copper nitrate trihydrate (90 mg, 0.37 mmol), 5-nitroisophthalic acid (79 mg, 0.37 mmol), 1,3-di-4-pyridylpropane (73 mg, 0.37 mmol) and 10.0 g water (550 mmol) was placed into a 23 ml Teflon-lined Parr Acid Digestion bomb, which was then heated under autogenous pressure at 363 K for 24 h. Blue blocks of (I) were obtained along with a light-blue amorphous powder.

### Refinement

All H atoms bound to C atoms were placed in calculated positions, with C—H = 0.95 - 0.99 Å, and refined in riding mode with *U*_iso_ = 1.2*U*_eq_(C). The H atom bound to the protonated carboxylate O atom was found in a difference Fourier map, restrained with O—H = 0.89 Å, and refined with *U*_iso_ = 1.2*U*_eq_(O).

The maximum and minimum residual electron density peaks of  1.10 and -0.31 e Å^-3^, respectively, were located 1.08 Å and 0.68 Å from the C14 and C17 atoms, respectively.

### Crystal data

**Table:**

[Cu(C_8_H_4_NO_6_)_2_(C_13_H_14_N_2_)]	*F*(000) = 1396
*M**_r_* = 682.05	*D*_x_ = 1.739 Mg m^−^^3^
Monoclinic, *C*2/*c*	Mo *K*α radiation, λ = 0.71073 Å
Hall symbol: -C 2yc	Cell parameters from 10349 reflections
*a* = 25.2976 (8) Å	θ = 1.8–25.4°
*b* = 5.3702 (2) Å	µ = 0.92 mm^−^^1^
*c* = 21.3122 (7) Å	*T* = 173 K
β = 115.865 (2)°	Block, blue
*V* = 2605.29 (15) Å^3^	0.22 × 0.22 × 0.11 mm
*Z* = 4	

### Data collection

**Table:**

Bruker APEXII diffractometer	2401 independent reflections
Radiation source: fine-focus sealed tube	2087 reflections with *I* > 2σ(*I*)
graphite	*R*_int_ = 0.032
ω and φ scans	θ_max_ = 25.4°, θ_min_ = 1.8°
Absorption correction: multi-scan (*SADABS*; Sheldrick, 1996)	*h* = −30→28
*T*_min_ = 0.821, *T*_max_ = 0.905	*k* = −6→6
10349 measured reflections	*l* = −24→25

### Refinement

**Table:**

Refinement on *F*^2^	Primary atom site location: structure-invariant direct methods
Least-squares matrix: full	Secondary atom site location: difference Fourier map
*R*[*F*^2^ > 2σ(*F*^2^)] = 0.041	Hydrogen site location: inferred from neighbouring sites
*wR*(*F*^2^) = 0.112	H atoms treated by a mixture of independent and constrained refinement
*S* = 1.08	*w* = 1/[σ^2^(*F*_o_^2^) + (0.0535*P*)^2^ + 8.8582*P*] where *P* = (*F*_o_^2^ + 2*F*_c_^2^)/3
2401 reflections	(Δ/σ)_max_ < 0.001
213 parameters	Δρ_max_ = 1.10 e Å^−^^3^
1 restraint	Δρ_min_ = −0.31 e Å^−^^3^

### Special details

**Table:**

Geometry. All e.s.d.'s (except the e.s.d. in the dihedral angle between two l.s. planes) are estimated using the full covariance matrix. The cell e.s.d.'s are taken into account individually in the estimation of e.s.d.'s in distances, angles and torsion angles; correlations between e.s.d.'s in cell parameters are only used when they are defined by crystal symmetry. An approximate (isotropic) treatment of cell e.s.d.'s is used for estimating e.s.d.'s involving l.s. planes.
Refinement. Refinement of *F*^2^ against ALL reflections. The weighted *R*-factor *wR* and goodness of fit *S* are based on *F*^2^, conventional *R*-factors *R* are based on *F*, with *F* set to zero for negative *F*^2^. The threshold expression of *F*^2^ > σ(*F*^2^) is used only for calculating *R*-factors(gt) *etc*. and is not relevant to the choice of reflections for refinement. *R*-factors based on *F*^2^ are statistically about twice as large as those based on *F*, and *R*- factors based on ALL data will be even larger.

### Fractional atomic coordinates and isotropic or equivalent isotropic displacement parameters (Å^2^)

**Table:**

	*x*	*y*	*z*	*U*_iso_*/*U*_eq_	Occ. (<1)
Cu1	0.0000	0.0000	0.0000	0.01865 (18)	
O1	0.07835 (8)	−0.1413 (4)	0.04676 (10)	0.0209 (5)	
O2	0.11395 (9)	0.1584 (4)	0.00388 (11)	0.0273 (5)	
O3	0.31946 (10)	0.1103 (5)	0.04659 (13)	0.0332 (6)	
H3A	0.3473 (12)	0.154 (7)	0.0365 (19)	0.040*	
O4	0.37815 (10)	−0.2063 (5)	0.10363 (13)	0.0374 (6)	
O5	0.30010 (10)	−0.7828 (4)	0.22279 (12)	0.0325 (6)	
O6	0.20769 (10)	−0.7829 (4)	0.19827 (11)	0.0279 (5)	
N1	0.01902 (10)	0.2420 (5)	0.07966 (12)	0.0207 (5)	
N2	0.25020 (11)	−0.6974 (5)	0.19249 (12)	0.0222 (6)	
C1	0.17873 (12)	−0.1639 (6)	0.07286 (14)	0.0180 (6)	
C2	0.22615 (12)	−0.0740 (6)	0.06286 (14)	0.0178 (6)	
H2	0.2213	0.0694	0.0348	0.021*	
C3	0.28055 (12)	−0.1922 (6)	0.09360 (14)	0.0189 (6)	
C4	0.28838 (12)	−0.3992 (6)	0.13549 (14)	0.0187 (6)	
H4	0.3252	−0.4823	0.1562	0.022*	
C5	0.24125 (13)	−0.4817 (5)	0.14633 (14)	0.0187 (6)	
C6	0.18627 (12)	−0.3712 (6)	0.11546 (14)	0.0178 (6)	
H6	0.1545	−0.4349	0.1231	0.021*	
C7	0.11985 (12)	−0.0359 (6)	0.03800 (14)	0.0190 (6)	
C8	0.33175 (12)	−0.0985 (6)	0.08267 (15)	0.0230 (7)	
C11	0.06442 (14)	0.2091 (7)	0.14297 (17)	0.0299 (7)	
H11	0.0897	0.0709	0.1492	0.036*	
C12	0.07641 (14)	0.3641 (7)	0.19921 (16)	0.0300 (8)	
H12	0.1088	0.3305	0.2428	0.036*	
C13	0.04037 (13)	0.5719 (6)	0.19170 (16)	0.0255 (7)	
C14	−0.00464 (13)	0.6150 (6)	0.12500 (16)	0.0270 (7)	
H14	−0.0290	0.7579	0.1164	0.032*	
C15	−0.01359 (13)	0.4504 (6)	0.07206 (16)	0.0265 (7)	
H15	−0.0445	0.4844	0.0274	0.032*	
C16	0.05196 (14)	0.7391 (7)	0.25328 (16)	0.0297 (7)	
H16A	0.0841	0.8549	0.2586	0.036*	
H16B	0.0662	0.6344	0.2957	0.036*	
C17	0.0000	0.8934 (9)	0.2500	0.0293 (10)	
H17A	−0.0137	1.0023	0.2084	0.035*	0.50
H17B	0.0137	1.0023	0.2916	0.035*	0.50

### Atomic displacement parameters (Å^2^)

**Table:**

	*U*^11^	*U*^22^	*U*^33^	*U*^12^	*U*^13^	*U*^23^
Cu1	0.0127 (3)	0.0208 (3)	0.0223 (3)	0.0016 (2)	0.0075 (2)	−0.0009 (2)
O1	0.0152 (9)	0.0220 (12)	0.0241 (10)	0.0019 (9)	0.0072 (8)	0.0005 (9)
O2	0.0225 (10)	0.0269 (13)	0.0338 (12)	0.0092 (10)	0.0135 (9)	0.0115 (10)
O3	0.0267 (12)	0.0337 (14)	0.0471 (14)	0.0025 (11)	0.0235 (11)	0.0139 (12)
O4	0.0198 (11)	0.0438 (15)	0.0491 (14)	0.0036 (11)	0.0153 (10)	0.0089 (12)
O5	0.0296 (12)	0.0306 (14)	0.0346 (12)	0.0131 (10)	0.0116 (10)	0.0145 (11)
O6	0.0330 (12)	0.0236 (12)	0.0294 (11)	−0.0015 (10)	0.0158 (10)	0.0040 (10)
N1	0.0171 (12)	0.0233 (14)	0.0213 (12)	0.0037 (11)	0.0079 (10)	0.0006 (11)
N2	0.0278 (14)	0.0191 (14)	0.0196 (12)	0.0036 (11)	0.0103 (11)	0.0000 (10)
C1	0.0160 (13)	0.0193 (15)	0.0174 (13)	0.0025 (12)	0.0061 (11)	−0.0026 (12)
C2	0.0197 (14)	0.0177 (15)	0.0157 (13)	0.0016 (12)	0.0075 (11)	0.0016 (11)
C3	0.0177 (14)	0.0212 (16)	0.0172 (13)	0.0008 (12)	0.0071 (11)	−0.0022 (12)
C4	0.0152 (13)	0.0203 (15)	0.0173 (13)	0.0032 (12)	0.0039 (11)	−0.0017 (12)
C5	0.0223 (14)	0.0176 (15)	0.0139 (13)	0.0015 (12)	0.0059 (11)	0.0002 (12)
C6	0.0161 (13)	0.0194 (16)	0.0173 (13)	−0.0022 (12)	0.0066 (11)	−0.0038 (12)
C7	0.0169 (14)	0.0216 (17)	0.0158 (13)	0.0018 (12)	0.0046 (11)	−0.0030 (12)
C8	0.0175 (14)	0.0279 (17)	0.0222 (15)	−0.0001 (13)	0.0072 (12)	−0.0001 (13)
C11	0.0274 (16)	0.0300 (19)	0.0332 (17)	0.0041 (14)	0.0142 (14)	0.0028 (15)
C12	0.0304 (17)	0.032 (2)	0.0257 (16)	−0.0019 (15)	0.0105 (13)	0.0022 (14)
C13	0.0227 (15)	0.0277 (18)	0.0283 (16)	−0.0034 (13)	0.0129 (13)	0.0012 (14)
C14	0.0239 (15)	0.0255 (18)	0.0327 (17)	−0.0002 (14)	0.0133 (13)	0.0007 (14)
C15	0.0217 (15)	0.0339 (19)	0.0236 (15)	0.0000 (14)	0.0098 (13)	0.0031 (14)
C16	0.0268 (17)	0.034 (2)	0.0261 (16)	−0.0030 (15)	0.0092 (13)	−0.0004 (14)
C17	0.033 (2)	0.030 (3)	0.025 (2)	0.000	0.0131 (19)	0.000

### Geometric parameters (Å, °)

**Table:**

Cu1—O1	1.9427 (19)	C3—C8	1.500 (4)
Cu1—O1^i^	1.9427 (19)	C4—C5	1.383 (4)
Cu1—N1^i^	2.022 (2)	C4—H4	0.9500
Cu1—N1	2.022 (2)	C5—C6	1.386 (4)
O1—C7	1.276 (3)	C6—H6	0.9500
O2—C7	1.243 (4)	C11—C12	1.380 (5)
O3—C8	1.318 (4)	C11—H11	0.9500
O3—H3A	0.85 (4)	C12—C13	1.405 (5)
O4—C8	1.206 (4)	C12—H12	0.9500
O5—N2	1.229 (3)	C13—C14	1.399 (4)
O6—N2	1.224 (3)	C13—C16	1.510 (5)
N1—C11	1.349 (4)	C14—C15	1.372 (5)
N1—C15	1.358 (4)	C14—H14	0.9500
N2—C5	1.472 (4)	C15—H15	0.9500
C1—C2	1.393 (4)	C16—C17	1.529 (4)
C1—C6	1.396 (4)	C16—H16A	0.9900
C1—C7	1.509 (4)	C16—H16B	0.9900
C2—C3	1.392 (4)	C17—C16^ii^	1.530 (4)
C2—H2	0.9500	C17—H17A	0.9900
C3—C4	1.385 (4)	C17—H17B	0.9900
			
O1—Cu1—O1^i^	180.00 (11)	O2—C7—C1	120.7 (3)
O1—Cu1—N1^i^	89.75 (9)	O1—C7—C1	115.1 (3)
O1^i^—Cu1—N1^i^	90.25 (9)	O4—C8—O3	124.6 (3)
O1—Cu1—N1	90.25 (9)	O4—C8—C3	123.3 (3)
O1^i^—Cu1—N1	89.75 (9)	O3—C8—C3	112.1 (2)
N1^i^—Cu1—N1	180.0	N1—C11—C12	124.0 (3)
C7—O1—Cu1	118.18 (18)	N1—C11—H11	118.0
C8—O3—H3A	112 (3)	C12—C11—H11	118.0
C11—N1—C15	115.8 (3)	C11—C12—C13	119.6 (3)
C11—N1—Cu1	122.9 (2)	C11—C12—H12	120.2
C15—N1—Cu1	121.3 (2)	C13—C12—H12	120.2
O6—N2—O5	123.6 (3)	C14—C13—C12	116.5 (3)
O6—N2—C5	118.5 (2)	C14—C13—C16	123.2 (3)
O5—N2—C5	117.9 (2)	C12—C13—C16	120.2 (3)
C2—C1—C6	119.6 (3)	C15—C14—C13	119.9 (3)
C2—C1—C7	119.9 (3)	C15—C14—H14	120.1
C6—C1—C7	120.6 (2)	C13—C14—H14	120.1
C3—C2—C1	120.7 (3)	N1—C15—C14	124.0 (3)
C3—C2—H2	119.7	N1—C15—H15	118.0
C1—C2—H2	119.7	C14—C15—H15	118.0
C4—C3—C2	120.2 (3)	C13—C16—C17	116.5 (2)
C4—C3—C8	118.6 (3)	C13—C16—H16A	108.2
C2—C3—C8	121.2 (3)	C17—C16—H16A	108.2
C5—C4—C3	118.3 (3)	C13—C16—H16B	108.2
C5—C4—H4	120.9	C17—C16—H16B	108.2
C3—C4—H4	120.9	H16A—C16—H16B	107.3
C4—C5—C6	122.9 (3)	C16—C17—C16^ii^	114.4 (4)
C4—C5—N2	118.1 (3)	C16—C17—H17A	108.7
C6—C5—N2	118.9 (3)	C16^ii^—C17—H17A	108.7
C5—C6—C1	118.3 (3)	C16—C17—H17B	108.7
C5—C6—H6	120.9	C16^ii^—C17—H17B	108.7
C1—C6—H6	120.9	H17A—C17—H17B	107.6
O2—C7—O1	124.2 (3)		
			
N1^i^—Cu1—O1—C7	94.8 (2)	Cu1—O1—C7—C1	−176.93 (17)
N1—Cu1—O1—C7	−85.2 (2)	C2—C1—C7—O2	−4.5 (4)
O1—Cu1—N1—C11	−17.8 (2)	C6—C1—C7—O2	175.1 (3)
O1^i^—Cu1—N1—C11	162.2 (2)	C2—C1—C7—O1	175.8 (2)
O1—Cu1—N1—C15	161.9 (2)	C6—C1—C7—O1	−4.6 (4)
O1^i^—Cu1—N1—C15	−18.1 (2)	C4—C3—C8—O4	6.5 (5)
C6—C1—C2—C3	1.5 (4)	C2—C3—C8—O4	−173.9 (3)
C7—C1—C2—C3	−178.9 (3)	C4—C3—C8—O3	−174.8 (3)
C1—C2—C3—C4	−1.0 (4)	C2—C3—C8—O3	4.8 (4)
C1—C2—C3—C8	179.4 (3)	C15—N1—C11—C12	4.0 (5)
C2—C3—C4—C5	−0.8 (4)	Cu1—N1—C11—C12	−176.3 (2)
C8—C3—C4—C5	178.9 (3)	N1—C11—C12—C13	−0.7 (5)
C3—C4—C5—C6	2.1 (4)	C11—C12—C13—C14	−3.1 (5)
C3—C4—C5—N2	−178.3 (2)	C11—C12—C13—C16	178.3 (3)
O6—N2—C5—C4	−174.7 (3)	C12—C13—C14—C15	3.4 (5)
O5—N2—C5—C4	4.8 (4)	C16—C13—C14—C15	−178.0 (3)
O6—N2—C5—C6	5.0 (4)	C11—N1—C15—C14	−3.6 (4)
O5—N2—C5—C6	−175.5 (3)	Cu1—N1—C15—C14	176.7 (2)
C4—C5—C6—C1	−1.5 (4)	C13—C14—C15—N1	−0.1 (5)
N2—C5—C6—C1	178.8 (2)	C14—C13—C16—C17	24.3 (5)
C2—C1—C6—C5	−0.3 (4)	C12—C13—C16—C17	−157.2 (3)
C7—C1—C6—C5	−179.9 (2)	C13—C16—C17—C16^ii^	62.0 (2)
Cu1—O1—C7—O2	3.4 (4)		

Symmetry codes: (i) −*x*, −*y*, −*z*; (ii) −*x*, *y*, −*z*+1/2.

### Hydrogen-bond geometry (Å, °)

**Table:**

*D*—H···*A*	*D*—H	H···*A*	*D*···*A*	*D*—H···*A*
O3—H3A···O2^iii^	0.85 (4)	1.86 (2)	2.668 (3)	158 (4)

Symmetry codes: (iii) −*x*+1/2, −*y*+1/2, −*z*.
